# A new cave-dwelling blind loach, Triplophysa erythraea sp. nov. (Cypriniformes: Nemacheilidae), from Hunan Province, China

**DOI:** 10.24272/j.issn.2095-8137.2019.049

**Published:** 2019-07-18

**Authors:** Tai-Fu Huang, Pei-Ling Zhang, Xing-Long Huang, Tao Wu, Xiao-Yan Gong, You-Xiang Zhang, Qing-Zhong Peng, Zhi-Xiao Liu

**Affiliations:** 1 College of Biology and Environmental Sciences, Jishou University, Jishou Hunan 416000, China

**Keywords:** *Triplophysa erythraea***sp. nov.**, Karst cave, Blind fish, Cyt *b*

## Abstract

A new blind loach species, Triplophysa erythraea sp. nov., from a karst cave in Hunan Province, central south China, is described based on morphology and cyt b gene sequencing. It can be distinguished from other species of Triplophysa by the following combination of characters: eyes absent; body scaleless and colorless; caudal-fin 17; maxillary barbel longest; fins transparent, compressed pectoral-fin reaching 2/3 distance between pectoral-fin and pelvic-fin origins; pelvic-fin and dorsal-fin origins relative; posterior chamber of airbladder well developed, long, oval, and dissociative.

Blindfish possess distinctly degenerated or completely lost eyes due to their cave or subterranean water system or deep ocean habitats (Zhao & Zhang, 2009). These specialized fish species are found within Cypriniformes, mostly belonging to *Sinocyclocheilus* or *Triplophysa*, and inhabit various inland river systems in China (Zhang & Dai, 2010). The genus *Triplophysa* (Nemacheilidae, Cypriniformes) is comprised of small fish living in streams and primarily distributed in the Tibetan Plateau and adjacent areas (He et al., 2011; Jacobsen et al., 2017; Xiao & Dai, 2011). A total of 202 species have been reported within the genus (Eschmeyer et al., 2018), including a number of cave-dwelling species (or populations) specialized for living in underground environments (Lan et al., 2013; Li et al., 2008; Yan, 2017). So far, a total of 30 cave-dwelling species of *Triplophysa* are known to occur in China (Li et al., 2017, 2018; Wu et al., 2018; Yan, 2017), of which 27 species have been identified from Yunnan, Guizhou, and Guangxi, and two from Chongqing (*T. rosa*) and Hunan (*T. xiangxiensis*) (Lan et al., 2013; Li et al., 2017; Wu et al., 2018; Yan, 2017; Zhang & Zhao, 2016; Figure 1).

In this paper, we describe a new species of cave-dwelling *Triplophysa* found during our exploration of cave animals in the Xiangxi Tujia and Miao Nationality Autonomous Prefecture, Hunan Province, central south China.

Specimens were collected in Dalong Cave (N28°16'25.11'', E109°28'57.18'', 563 m a.s.l.), Huayuan County, Hunan Province, central southern China. Morphometric measurements were made on fresh specimens with a digital caliper (0.01 mm) and a stereomicroscope (XTL-165VT) in accordance with the protocols described in the literature (Kottelat, 1984; Zhu, 1989). An abdominal muscle sample was removed and placed in ethanol (70%) for genome DNA extraction. The specimens were then preserved in formalin (10%) and deposited in the Zoological Collection Room, College of Biology and Environmental Sciences, Jishou University.

DNA extraction and mitochondrial cytochrome *b* (cyt *b*) gene sequencing were carried out as described previously (Yan, 2017). Samples were sequenced by Sangon Biotech (Shanghai, China) using the Sanger method on an ABI 3707 (ABI, USA) instrument. The sequencing results were uploaded to GenBank after the Blast program was run in the NCBI database for confirmation, and the cyt *b* sequences of several approximate species were downloaded for molecular analysis. Genetic distances were calculated by Mega 7.0 (Kumar et al., 2016) based on the Kimura 2-parameter (K2p) model (Kimura, 1980).

**Figure 1:**
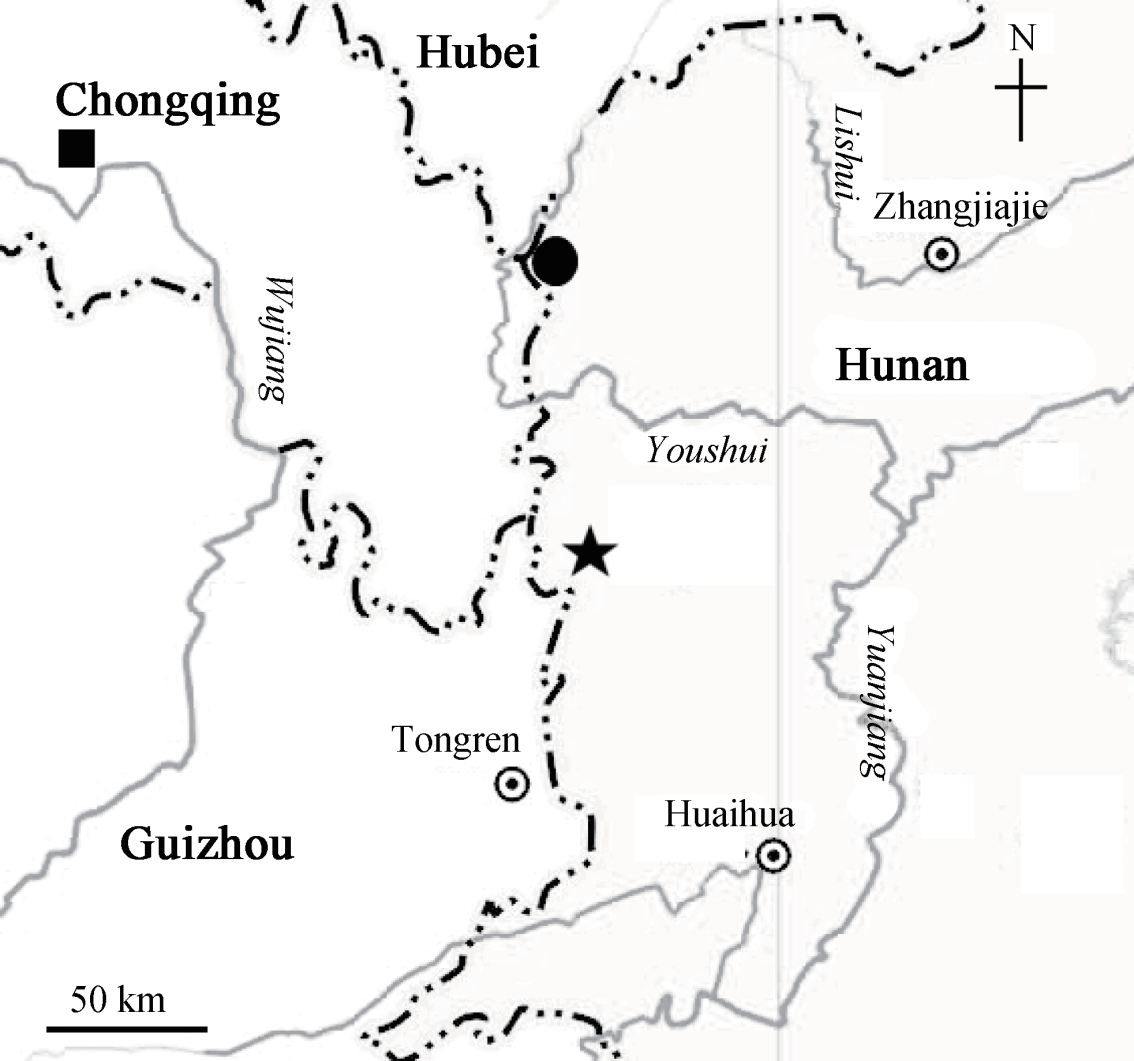
**Distribution of *Triplophysa erythraea *sp. nov. (**★**), *T. xiangxiensis* (**●**), and *T. rosa* (**■**)**

**Figure 2:**
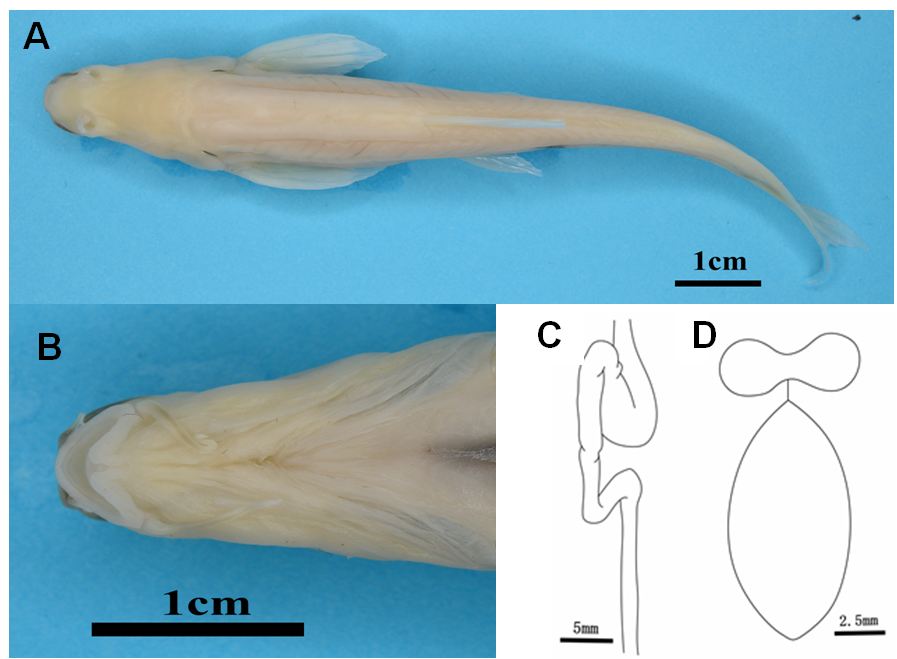
Morphology of *Triplophysa erythraea* sp. nov. A: Dorsal view; B: Ventral view of head; C: Exterior view of digestive duct; D: Ventral view of bony capsule and rearward airbladder.

**Figure 3:**
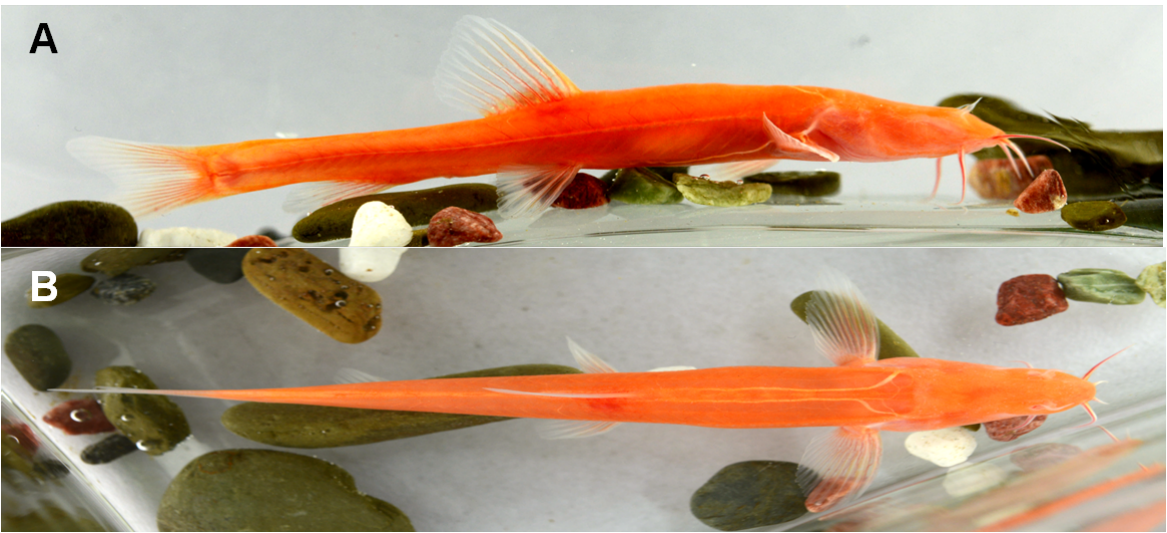
Live specimens of *Triplophysa erythraea *sp. nov.

**Table 1:** Morphometric data of *Triplophysa erythraea *sp. nov. (all in mm)

Item	Holotype	Paratypes		Mean±*SD*
	HY18022802	HY18011301	HY18022801	
Total length (TL)	105.98	81.53	109.34	98.95±15.18
Standard length (SL)	88.22	68.49	88.58	81.76±11.50
Head length (HL)	23.44	18.49	23.44	21.79±2.86
Dorsal length	16.44	15.72	18.14	16.77±1.24
Pelvic length	13.40	10.74	13.4	12.51±1.54
Pectoral length	17.96	14.65	25.35	19.32±5.48
Anal length	13.72	9.67	14.73	12.71±2.68
Body depth/(SL) (%)	13.59	13.00	16.37	14.32±1.80
Predorsal length/(SL) (%)	53.63	52.62	55.14	53.80±1.27
Prepectoral length/(SL) (%)	28.71	26.19	28.71	27.87±1.45
Preanal length/(SL) (%)	76.91	75.61	76.91	76.48±0.75
Caudal-peduncle length/(SL) (%)	18.18	16.67	18.18	17.68±0.87
Caudal-peduncle depth/(SL) (%)	7.73	7.73	9.18	8.21±0.84
Length of dorsal-fin base/(SL) (%)	11.41	11.32	12.77	11.83±0.81
Length of anal-fin base/(SL) (%)	7.89	6.29	7.87	7.35±0.92
Length of pectoral-fin base/(SL) (%)	5.07	4.53	5.07	4.89±0.31
Length of pelvic-fin base/(SL) (%)	2.45	2.45	7.45	4.12±2.89
Upper jaw length/(SL) (%)	5.60	5.60	6.69	5.96±0.63
Lower jaw length/(SL) (%)	4.55	4.34	5.00	4.63±0.34
Mouth width/(SL) (%)	6.69	6.69	7.78	7.05±0.63
Nose width/(HL) (%)	17.36	17.36	18.39	17.70±0.59
Outer rostral barbell length/(HL) (%)	27.22	27.22	40.18	31.54±7.48
Inner Rostral barbell length/(HL) (%)	20.69	16.66	22.17	19.84±2.85
Maxillary barbell length/(HL) (%)	31.53	31.53	43.19	35.42±6.73

## Taxonomy*Triplophysa erythraea*sp. nov. Liu & Huang

Figures 2, 3; Table 1.

**Etymology:** The specific name “*erythraea*” is derived from the blood-red body color of the living fish. Although their body surface is colorless and transparent, the red blood vessels are visible.

**Holotype:** HY18022802, intact, undissected, 105.98 mm TL (total length), 88.22 mm SL (standard length); Dalong Cave, Huayuan County, Hunan, central southern China, 28 February 2018; coordinates N28°16'25.11'', E109°28'57.18'', 563 m a.s.l..

**Paratypes:** HY18011301, dissected, 81.53 mm TL, 68.49 mm SL, 13 January 2018; HY18022801, dissected, 109.34 mm TL, 88.58 mm SL, 28 February 2018; two specimens collected with holotype.

**Diagnosis:** *Triplophysa erythraea* **sp. nov.** can be distinguished from all species of *Triplophysa* by the following combination of characters: eyes absent; body scaleless and colorless; pectoral-fin ii-10, dorsal-fin ii-8, pelvic-fin ii-5, anal-fin i-6, caudal-fin 17; maxillary barbel longest; fins transparent, compressed pectoral-fin reaching 2/3 of distance between pectoral-fin and pelvic-fin origin; pelvic-fin and dorsal-fin origin relative; edge of compressed pelvic-fin reaching anus; caudal-fin forked; posterior chamber of airbladder well developed, long, oval, and dissociative.

**Description: External characteristics**: Morphometric data of the new species specimens are given in Table 1. Dorsal-fin ii-8, anal-fin i-6, pectoral-fin ii-10, pelvic-fin ii-5, caudal-fin 17, 11 gill rakers in inner row on first gill arch. Cephalic lateral-line canals with 4 supratemporal, 7–9 supraorbital, 2+9–11 infraorbital, and 12 preoperculo-mandibular pores. Lateral line complete, with 63–67 pores.

Body elongated, scaleless, body side compressed posteriorly; abdominal organs and vessels of barbel, fins, and body side visible. Eyes absent. Ventral view of head fusiform, dorsum of head slightly concave in middle, posterior trunk becoming gradually smaller; trailing edge of gill covers widest and highest point of body. Snout dull, mouth inferior, upper and lower jaw arched. Edge of jaws leathery, upper jaw more developed, dentate process in center of maxilla. Lips developed, smooth, papillary process absent, lower lip with V-type median notch.

Naris circular, slanting down; anterior and posterior naris close, diameter of posterior naris larger; anterior naris in elongated nose flap without barbel-like tip, nose flap developed as triangular petal. Three pairs of developed barbels; one pair of inner rostral barbels, outer rostral barbel, and maxillary barbel, respectively; maxillary barbel longest, outer rostral barbel longer than inner rostral barbel.

Fins transparent, pterygiophore clearly visible. Pectoral-fin compressed reaching 2/3 of distance between pectoral-fin and pelvic-fin origin, first branched ray not extended; dorsal-fin distally truncate, dorsal-fin compressed slightly longer than pelvic-fin. Distance from dorsal-fin origin to caudal-fin origin closer than to snout. Pelvic-fin origin and dorsal-fin origin equal, edge of pelvic-fin reaching anus. Distance from pelvic-fin origin to anal-fin origin and caudal-fin origin to anal-fin origin equidistant. Caudal-fin forked, lobe tip; anus close to anal-fin origin.

**Internal structure**: Four pairs of gill arches, gill filaments intensive. Anterior chamber of airbladder wrapped in dumbbell-shaped bony capsule; posterior chamber expanded, dissociative, long, and oval. Stomach enlarged and U-shaped. Back of intestine bent in Z-shape. Length of intestine shorter than body length, indicating that new species is a possible demersal carnivorous fish.

**Coloration:** Live adults of the new species are bright red in color (Figure 3A, B), which is related to their visible blood vessels, not by skin pigmentation. Larvae are reddish white. Adults fixed in 10% formalin are pale (Figure 2A).

**Sexual dimorphism:** No sexual dimorphism was observed in the specimens examined.

**Figure 4:**
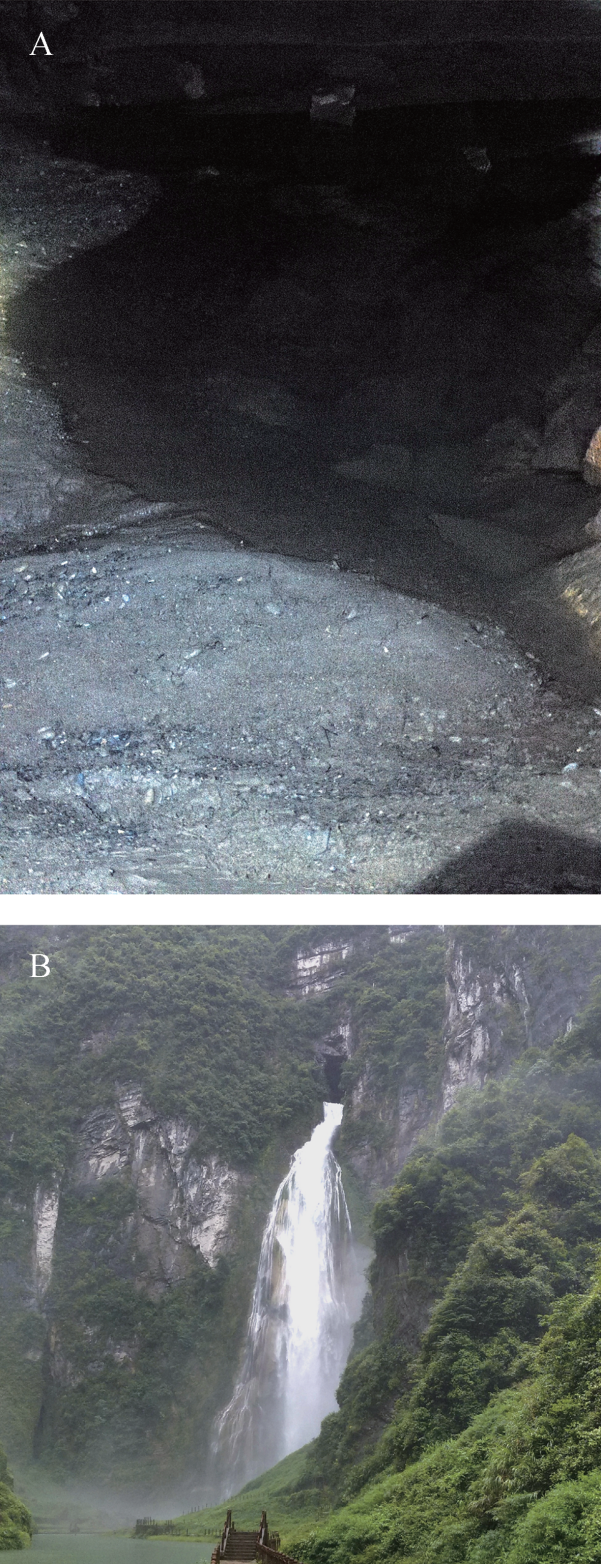
Habitat of *Triplophysa erythraea *sp. nov. A: Dalong cave pool where specimens were collected; B: Waterfall at cave entrance during rainy season.

**Distribution and habitat:** The new species is known only from Dalong Cave, Huayuan County, Hunan Province, China (Figure 1). At the cave entrance is a hydroelectric hub, from which rushes out a fast-flowing underground river (Figure 4). The new species was found 200–450 m into the cave tunnel from the entrance, with a mean water temperature and pH of 13.5 °C and 6.0, respectively. A total of 14 blind fish (including 12 adults and two larvae) were found in the resting shallow waters during our two surveys. We did not reach the end of the cave during the surveys due to the presence of an underground river. Additional research would be beneficial to understand the eco-biological characteristics of this new species in threatened conditions.

**Molecular analysis:** The length of the cyt *b* gene sequence was 1 140 bp (GenBank accession No.: MG967615), and the average content of the A, T, C, and G bases was 28.2%, 28.2%, 28.2%, and 15.4%, respectively. NCBI Blast analysis showed that the new species had highest similarity (90%) to *T. lewangensis*, although their genetic distances was 11.9%, (Table 2), which surpassed the interspecies-averaged genetic distance (11.5%) of cave-dwelling *Triplophysa* based on the cyt *b* gene (Yan, 2017).

**Table 2:** K2p genetic distances based on the cyt *b* gene between *Triplophysa erythraea *sp. nov. and approximate species of *Triplophysa*

No.	Species (GenBank accession No.)	1	2	3
1	New species (MG967615)			
2	*T.lewangensis* (KU987438)	0.119		
3	*T. xiangxiensis* (KT751089)	0.127	0.089	
4	*T. rosa* (JF268621)	0.131	0.099	0.096

Although eyeless, with a scaleless and colorless body, the new species displayed a highly developed lateral line and barbel, indicating that it was a typical cavefish (Zhang & Dai, 2010; Zhao & Zhang 2006). Furthermore, it was easily distinguished from other species in the genus *Triplophysa* due to differences in eye normality or degeneration into a small black dot. Eyeless species in *Triplophysa* include *T. gejiuensis* (Chu & Chen, 1979), *T. xiangxiensis* (Yang et al., 1986), *T. shilinensis* (Chen et al*.*, 1992*)*, *T. longibarbatus* (Chen et al*.*, 1998), *T. qiubeiensis* (Li et al., 2008), *T. huanjiangensis* (Yang et al., 2011), *T. jiarongensis* (Lin et al., 2012), *T. lihuensis* (Wu et al., 2012), *T. fengshanensis* (Lan et al., 2013), *T. dongganensis* (Lan et al., 2013), and *T. anshuiensis* (Wu et al., 2018).

The new species can be distinguished from *T.xiangxiensis*, *T. longibarbatus*, and *T. jiarongensis* by the following characters: compressed pectoral-fin reaching 2/3 of distance between pectoral-fin and pelvic-fin origin (vs*.* compressed pectoral-fin reaching or exceeding origin of pelvic-fin). It can be distinguished from *T. lihuensis*, *T. fengshanensis*, and *T. dongganensis* by the following characters: maxillary barbel longest, caudal-fin forked (vs*.* outer rostral barbel longest, caudal-fin shallow). The new species can be distinguished from *T. gejiuensis* by the following characters: distance from dorsal-fin origin to caudal-fin origin closer than to snout; maxillary barbel longest (vs*.* distance from dorsal-fin origin to snout closer than to caudal-fin origin; outer rostral barbel longest). It can be distinguished from *T. shilinensis* by the following characters: head tapered, front of dorsal-fin slightly compressed; posterior chamber of airbladder well developed; caudal-fin 17 (vs*.* head long and pointed, front of dorsal-fin slightly cylindrical; posterior chamber of airbladder degenerated; caudal-fin 14). It can be distinguished from *T. huanjiangensis* by the following characters: lip smooth, papillary process absent; pelvic-fin origin relative to vertical line of dorsal-fin origin; lateral line complete (lip papillary process present; pelvic-fin origin anterior to vertical line of dorsal-fin origin; lateral line absent). It can be distinguished from *T. qiubeiensis* by the following characters: lip developed, smooth, papillary process absent; dentate process in center of maxilla; dorsal-fin distally truncate; posterior chamber of airbladder well developed (vs*.* lip developed, smooth, papillary process present; no dentate process in center of maxilla; dorsal-fin distally truncate; posterior chamber of airbladder degenerated). It can be distinguished from *T. anshuiensis* by the following characters: maxillary barbel longest; pigments absent (vs. inner rostral barbel longest; black pigments irregularly present on dorsum of body).

The genetic distances indicated that the new species was most closely related to *T. lewangensis*, although the values surpassed the interspecies-averaged genetic distances (Yan, 2017) and *T. lewangensis* possess eyes (Liang & Zhou, 2019). In conclusion, based on morphology and cyt *b* gene sequencing, the blind loach collected from Dalong Cave (N28°16'25.11'', E109°28'57.18''), Hunan Province, central south China, was designated as a new species: *Triplophysa erythraea* **sp. nov.**

In particular, the live adult coloration (bright-red) of the new species was different from that of other cave-dwelling *Triplophysa* species (whitish, pink, or gill cover red). The collected individuals of the new species were fed for one month in our laboratory and did not change body color during that time. This suggests that the new species should be formally designated as albino; however, further research is required to determine whether the pigment regulator genes were lost.

## NOMENCLATURAL ACTS REGISTRATION

The electronic version of this article in portable document format will represent a published work according to the International Commission on Zoological Nomenclature (ICZN), and hence the new names contained in the electronic version are effectively published under that Code from the electronic edition alone (see Articles 8.5–8.6 of the Code). This published work and the nomenclatural acts it contains have been registered in ZooBank, the online registration system for the ICZN. The ZooBank LSIDs (Life Science Identifiers) can be resolved and the associated information can be viewed through any standard web browser by appending the LSID to the prefixhttp: //zoobank.org/.Publication LSID:urn: lsid: zoobank.org: pub: 1F4D3CF7-3676-4EDC-B4F9-75F10FEA8F90.Nomenclatural act LSID:urn: lsid: zoobank.org: act: 8D92F37F-DB60-4395-95F1-09F0410ABAB1.
